# A WeChat-based Intervention, Wellness Enhancement for Caregivers (WECARE), for Chinese American Dementia Caregivers: Pilot Assessment of Feasibility, Acceptability, and Preliminary Efficacy

**DOI:** 10.2196/42972

**Published:** 2023-04-05

**Authors:** Y Alicia Hong, Kang Shen, Hae-Ra Han, Van Ta Park, Pramita Bagchi, Huixing Kate Lu, Hsiaoyin Chen, Judy Huei-yu Wang

**Affiliations:** 1 Department of Health Administration and Policy College of Public Health George Mason University Fairfax, VA United States; 2 School of Nursing Johns Hopkins University Baltimore, MD United States; 3 Department of Community Health Systems School of Nursing University of California San Francisco San Francisco, CA United States; 4 Department of Statistics George Mason University Fairfax, VA United States; 5 Chinese Culture and Community Service Center, Inc Gaithersburg, MD United States; 6 Department of Oncology Lombardi Comprehensive Cancer Center Georgetown University Washington, DC United States

**Keywords:** Alzheimer disease, dementia, caregiver, Chinese American, mHealth intervention, social media, WeChat, mHealth, mobile health, informal care, caregiving, family care, spousal care, minority, ethnic, cultural, Chinese

## Abstract

**Background:**

Chinese American family caregivers of persons with dementia experience high rates of psychosocial distress and adverse health outcomes. Due to their immigrant and minority status, they face substantial obstacles to care and support, including stigma and misperception of dementia, limited knowledge and use of welfare and services, and poor social support. Few interventions have been developed or tested for this vulnerable population.

**Objective:**

This study aims to pilot-test the Wellness Enhancement for Caregivers (WECARE) intervention, a culturally tailored program delivered via WeChat, a social media app highly popular in the Chinese population. The 7-week WECARE was designed specifically for Chinese American dementia caregivers to improve their caregiving skills, reduce stress, and enhance psychosocial well-being. Feasibility, acceptability, and preliminary efficacy of the WECARE were assessed in this pilot.

**Methods:**

A total of 24 Chinese American family caregivers of persons with dementia were recruited for a pre-post 1-arm trial of the WECARE. By subscribing to the WECARE official account, participants received interactive multimedia programs on their WeChat account multiple times a week for 7 weeks. A backend database automatically delivered program components and tracked user activities. Three online group meetings were organized to facilitate social networking. Participants completed a baseline and a follow-up survey. Feasibility was assessed by the follow-up rate and curriculum completion rate; acceptability was assessed by user satisfaction and perceived usefulness of the program; and efficacy was assessed with pre-post differences in 2 primary outcomes of depressive symptoms and caregiving burden.

**Results:**

The intervention was completed by 23 participants with a retention rate of 96%. Most of them (n=20, 83%) were older than 50 years and the majority (n=17, 71%) were female. The backend database revealed that the mean curriculum completion rate was 67%. Participants also reported high rates of user satisfaction and perceived usefulness of the intervention and high ratings of weekly programs. The intervention led to significant improvement in participants’ psychosocial health outcomes; their depressive symptoms reduced from 5.74 to 3.35 with an effect size of −0.89 and caregiving burden decreased from 25.78 to 21.96 with an effect size of −0.48.

**Conclusions:**

This pilot study suggests that WeChat-based WECARE intervention was feasible and acceptable; it also demonstrated initial efficacy in improving psychosocial well-being in Chinese American dementia caregivers. Further research with a control group is needed to assess its efficacy and effectiveness. The study highlights the need for more culturally appropriate mobile health interventions for Chinese American family caregivers of persons with dementia.

## Introduction

The American population is rapidly aging and becoming more diverse. The number of adults aged 65 years and older is projected to increase from 16.9% in 2020 to 22% in 2050, and the proportion of racial and ethnic minorities is expected to grow from 38.4% in 2020 to 50.3% in 2045 [1]. Currently, there are 6 million persons with dementia and 19 million family caregivers of persons with dementia in the United States. These numbers are expected to double by 2050 due to aging of the population [2].

Family caregivers of persons with dementia face a myriad of challenges related to the length, complexity, and intensity of caregiving. As a result, they have reported high levels of physical and emotional stress [3,4], depression and anxiety [5-7], poorer quality of sleep [8,9], and poorer quality of life [10-12]. Some develop chronic conditions including impaired immune functions, hypertension, and coronary health diseases [13]. Despite high rates of psychosocial distress in dementia caregivers, the existing interventions for racial or ethnic minority dementia caregivers are rather limited compared to those available to the White population [14].

Asian Americans are the fastest-growing racial group with a growth rate of 88% from 2000 to 2020 [15]. Chinese Americans make up 23% of the Asian American population with nearly 70% of them being foreign-born [16]. To date, the literature on Chinese American dementia caregivers is limited and mostly descriptive. Available literature suggests that Chinese American dementia caregivers face triple challenges. First, their perceptions of dementia, appraisals of stress, coping, and help-seeking behaviors are shaped by Chinese cultural norms of “filial piety” and “family harmony” [17]. Caring for older family members is not only a sign of love and pride but also a moral obligation [18,19]. Adherence to such cultural values helps caregivers find positive aspects of caregiving and also leads to psychosocial stress when perceived performance or outcomes could not match their filial expectations [20,21]. Under the “family harmony” cultural values, caregivers are more likely to internalize stress and less likely to seek external help. Second, as most Chinese American family caregivers are foreign-born, their immigration status and unfamiliarity with the health care and welfare systems render limited knowledge and use of formal services [18,22,23]. Third, compared to long-time residents, first-generation immigrants have smaller social networks, less buffer zones, and limited social support. Chinese American family caregivers with limited English proficiency face additional linguistic and cultural barriers and are more socially isolated [24,25].

Despite these unique challenges and barriers, few caregiving interventions are tailored to the needs of Chinese American dementia caregivers [26]. In a recent review of global literature on existing interventions for Chinese dementia caregivers, only 2 were designed specifically for Chinese Americans [27]. One was a home-based behavioral management program and the other was a DVD-based psychoeducation program [28,29]. Both pilot interventions, developed by Gallagher-Thompson and colleagues [28,29] in early 2000s, cannot meet the current needs of Chinese American dementia caregivers. A strong need exists for easy-to-access and easy-to-scale mobile health (mHealth) intervention for this vulnerable population. This need has become more salient since the COVID-19 pandemic when in-person contact was restricted and the need for remote services was increasing.

Racial or ethnic minority populations are more likely to be smartphone-dependent for internet access (without computer or other mobile devices) and rely on social media as a primary source of health information [30]. With a 90% penetration rate in Chinese-speaking Chinese Americans, WeChat is the most popular social media app in this population [31]. The key functions of WeChat include “moments” for sharing photos and stories with friends and receiving “likes” and feedback, texting, voice call, video call, private chat, group chat, location sharing, file transfer, and payment. These built-in functions allow intervention developers to focus on program content rather than technical aspects of development maintenance, thus saving time and cost. It also enables easy adoption and long-term use, especially in populations with lower levels of health literacy [32]. Literature has shown the feasibility, acceptability, efficacy, and even long-term effectiveness of WeChat-based interventions in Chinese populations [33-36]. Thus, a popular and versatile social media app like WeChat could serve as a viable delivery channel for mHealth interventions to reach Chinese American dementia caregivers.

To address the literature gaps and public health needs of culturally tailored intervention for Chinese American dementia caregivers, we developed a WeChat-based intervention called Wellness Enhancement for Caregivers (WECARE) to address their psychosocial distress [37]. This paper reports the results from piloting WECARE, including its feasibility, acceptability, and initial efficacy.

## Methods

### Overview

This is a 1-arm, pre-post pilot trial. A total of 24 Chinese American dementia caregivers completed a baseline survey and received the 7-week WECARE intervention; their activities on WECARE were tracked by the backend database. Participants completed a follow-up survey 2 to 3 weeks after the intervention. Feasibility was assessed by the retention rate and curriculum completion rate. Acceptability was assessed by user satisfaction and perceived usefulness scale in the follow-up survey. Preliminary efficacy was evaluated by effect sizes of psychosocial health outcomes assessed at baseline and follow-up surveys.

### Participation Eligibility

Participation eligibility included (1) self-identifying as Chinese or Chinese Americans and can read Chinese, (2) at least 21 years old, (3) currently living in the United States, (4) using WeChat, and (5) providing care at least 12 hours a week for a family member or loved one with Alzheimer disease, dementia, or other neurodegenerative conditions. Exclusion criteria included (1) being cognitively impaired or has serious mental health problems and (2) care partner has less than 6 months of life expectancy or in hospice care. When a potential participant contacted, our research staff conducted the screening. Those who met the participation criteria were invited to participate. A separate Zoom meeting was scheduled to obtain informed consent.

### Recruitment

Participants were recruited from 2 sources. One was through our community partner, a community-based organization that serves Chinese Americans in the greater Washington, DC metropolitan. A recruitment flyer was distributed through social media and email newsletters. The other source was the University of California San Francisco Collaborative Approach for Asian Americans, Native Hawaiians, and Pacific Islanders Research Education registry [38]. Potentially interested participants contacted a designated phone number for more information and screening. Those who met the participation eligibility would learn more about the study and be invited to participate. A separate Zoom meeting would be scheduled for informed consent. Participants who completed the 7-week intervention plus the baseline and follow-up surveys would receive a gift card of US $100.

### Ethics Approval

The study protocol was approved by the Institutional Review Board of George Mason University (IRB#1849712). All eligible participants had a one-on-one online meeting with a research staff who explained the study procedure and answered all questions. All participants provided verbal informed consent before they started the study.

### WECARE Intervention

The 7-week WECARE intervention was developed to reduce caregiving burden, decrease distress, and enhance psychosocial well-being of Chinese American family caregivers of persons with dementia. Its protocol development and key features were detailed elsewhere [37]. By subscribing to the WECARE official account, participants would receive 6 multimedia articles on their WeChat accounts each week for the first 6 weeks and 4 in the final week for a total of 40 articles. Each week was focused on a theme. The seven major themes included (1) facts and knowledge of dementia and caregiving; (2) enhancement of caregiving skills; (3) effective communication with health care providers, care partners, and family members; (4) problem-solving skills for caregiving stress management; (5) stress reduction and depression prevention; (6) practice of self-care and health behaviors; and (7) social support and local resources. All multimedia articles required 3 to 6 minutes read time. Embedded in the articles were pictures, short video clips, and downloadable forms; all articles were culturally tailored for the target population and accompanied by audio recordings in case some older caregivers would prefer listening to audio recordings over reading texts. Three moderated group meetings were organized at weeks 3, 5, and 7 to facilitate social networking. Participants could also use the built-in functions in WeChat to initiate group chats, private chats, or video calls for additional networking and peer support. The official account of WECARE had a backend database that managed intervention delivery and tracked user activities [37].

### Intervention Delivery and Data Collection Procedure

A total of 24 participants were enrolled in the study. After the informed consent, participants completed a web-based baseline survey and then subscribed to the WECARE official account via their WeChat app. WECARE automatically sent multimedia program components 4-6 times a week, at a prescheduled time Monday to Saturday, for 7 weeks. During the 7 weeks, participants’ activities on WECARE, including whether a program component was opened, how many times it was opened, and for how long, were tracked by the backend database. Participants who had not opened WECARE for a week would receive a friendly reminder via WeChat. Those who were not responsive to our reminders for 3 consecutive weeks were considered dropped out. A follow-up survey was administered 2 to 3 weeks after the intervention completion. Surveys were in Chinese, the links to the online surveys were sent to participants in their WeChat accounts or emails, and they could open the link in any browser.

### Measurement

#### Overview

Two sets of data were collected in the pilot study: (1) baseline and follow-up surveys administered online before and after the intervention (see Multimedia Appendix 1 for complete baseline and follow-up surveys) and (2) user activities tracked by the backend databases (see Multimedia Appendix 2 for screenshots of WECARE frontend and backend). These data were used to assess the feasibility, acceptability, and initial efficacy of the WECARE intervention. Table 1 illustrates the domains of measures and data sources.

**Table 1:** Data sets and domains of measures.

			Baseline survey		Backend database		Follow-up survey
Demographics			✓				
**Feasibility**							
	Retention rate	✓				✓	
	Curriculum completion rate			✓			
	User activities: total and weekly read counts; total and weekly reading minutes			✓			
**Acceptability**							
	User satisfaction					✓	
	Perceived usefulness of WECARE^a^					✓	
	Perceived usefulness of weekly program					✓	
**Efficacy**							
	Depressive symptoms	✓				✓	
	Caregiver’s burden	✓				✓	
	Life satisfaction	✓				✓	
	Perceived social support	✓				✓	

^a^WECARE: Wellness Enhancement for Caregivers.

#### Feasibility

Feasibility was measured by three indicators about how participants have completed the intervention trial: (1) retention rate was assessed by the percentage of participants who completed the follow-up survey. (2) Curriculum completion rate was assessed by the percentage of a participant’s completion of all 40 articles of the WECARE curriculum. For example, if a participant completed 20 articles, his or her curriculum completion rate was 50%. If an article was opened, it was considered read or completed, which was tracked by the backend database. We calculated the mean value of all curriculum completion rates of all participants. (3) User activity was assessed by read counts and reading minutes tracked by the backend database. A “read count” was the number of times a participant had opened an article; “weekly read count” was the sum of read counts on a week’s program; and “total read count” was the sum of all read counts. “Reading minutes” was the minutes a participant spent on an article; “weekly reading minutes” was the sum of reading minutes for a week’s program; and “total reading minutes” was the sum of all reading minutes. These indicators of user activity reflected user engagement.

#### Acceptability

Acceptability was measured by three indicators, all were drawn from our previous digital health intervention evaluation [39]. The first two were about their experience of the overall WECARE program; the last one was about each of the weekly program. (1) User satisfaction was assessed with a 7-item user-satisfaction in the follow-up survey on how participants liked the WECARE program, for example, “it was easy to use,” “it was fun to use,” and “I would recommend it to my friends or family.” Each question has response options from strongly disagree to strongly agree. The total score had a range of 5-35 with a higher score indicating a higher level of user satisfaction. The Cronbach α for the scale was .737. (2) Perceived usefulness of WECARE was assessed with a 5-item scale in the follow-up survey on how participants perceived the WECARE intervention had helped them, for example, “become a better caregiver” and “learn more about stress management and mental health.” Each item has 5 response options from strongly disagree to strongly agree. The total score had a range of 5-25 with a higher score indicating a higher level of perceived usefulness The Cronbach α for the scale was .834. (3) Perceived usefulness of weekly program: The follow-up survey included questions asking participants how useful the weekly programs and moderated group meetings were. The response options ranged from not useful at all (1) to very useful (5). The mean score was calculated for each weekly program and the group meetings.

#### Intervention Efficacy

Intervention efficacy was measured by whether the 4 psychosocial health outcomes have meaningful effect sizes as a result of the intervention. The primary outcomes were depressive symptoms and caregiver’s perceived burden, and the secondary outcomes were life satisfaction and perceived social support. (1) Depressive symptoms were assessed by the Center for Epidemiologic Depression Scale (CES-D) 10-item [40]. Participants were asked to rate whether they experienced symptoms associated with depression the past week (0=no and 1=yes) with a total score ranging from 0 to 10 with a clinical cutoff point of 4 as an indicator of elevated depressive symptoms [41]. The CES-D has been used to monitor and identify trajectories of depressive symptoms and has been validated with Chinese populations [42,43]. In this study, the Cronbach α for depressive symptoms at baseline was .809. (2) Caregiving burden was assessed by the Zarit Burden Interview (ZBI). The 12-item ZBI is one of the most reliable measures of caregiver burden in the literature. Each item has 5 response categories from “never” to “nearly always” with individual scores from 0 to 4 for each item [44]. Across the 12 items, the total ZBI score has a range of 0-48 with a cutoff point of 13 for community-dwelling caregivers [45]. ZBI has been validated in Chinese populations [46,47]. The Cronbach α for ZBI at baseline was .824. (3) Life satisfaction was assessed by the Satisfaction With Life Scale (SWLS) [48]. The SWLS contained 5 items and used a 7-point Likert-type scale from 1 (strongly disagree) to 7 (strongly agree). The SWLS assessed the individual’s evaluation of his or her life by using the person’s own criteria (eg, “In most ways, my life is close to my ideal”). It has been validated in Chinese older adults and Chinese dementia caregivers [49]. The Cronbach α for SWLS at baseline was .915. (4) Perceived social support was assessed by a 10-item scale adapted from Social Support Scale (SSC). Validated in the REACH II study, this scale used a 4-point Likert scale from 0=never, 1=occasionally, 2=sometime, and 3=always to assess how often caregivers receive social support from family or friends [50]. The total score of social support ranged from 0 to 30 with a higher score indicating a higher level of social support. The Cronbach α for SSC was .756.

#### Demographic Characteristics

Demographic characteristics of participants were assessed in the baseline survey. Caregivers’ characteristics assessed included age, sex, marital status, education, employment status, years of living in the United States, English proficiency, health status, and having difficulty paying for the basics. Care-partner characteristics assessed included age, sex, relationship to caregiver, living arrangement, and functional status measured by activity of daily living (ADL) [51] and instrumental activities of daily living (IADL) [52].

### Statistical Analysis

First, descriptive statistics were used to describe the sample characteristics, feasibility and user engagement, acceptability, and user satisfaction. Cronbach α was used to calculate internal consistency of the scales. Then, paired *t* test was used to compare pre-post differences in efficacy measures of psychosocial health outcome; the statistical significance was set as *P* value ≤.10. Finally, given the small sample size, we calculated effect sizes for the health outcomes [53]. The small sample size also limited the power for stratified analysis, so we did not conduct multivariate analysis to examine the independent relationship between the outcome variables and independent variables such as demographics and user engagement. All analyses were conducted using Stata (version 14; StataCorp).

## Results

### Participant Characteristics

As shown in Table 2, a total of 24 participants were enrolled in the study, 71% (n=17) were female, and 88% (n=21) were married or living with a partner. Their ages ranged from 38 to 85 years, with 83% (n=20) were older than 50 years of age, and the mean age was 60 (SD 11.99) years. All participants were born in China and had lived in the United States for 23 years on average (ranged 3 to 44 years). About 54% (n=13) had limited English proficiency, and 46% (n=11) had difficulty paying for the basics. Many caregivers (n=16, 67%) were taking care of their parents or parents in-law, 29% (n=7) were taking care of a spouse, and 1 was taking care of a friend. Care partners’ ages ranged from 60 to 91 years with a mean of 81 years. Care partners’ mean ADL score was 12 (ranged 0 to 27) and mean IADL score was 20 (ranged 7 to 24).

**Table 2:** Demographics of caregivers and care partners.

Characteristics				Values
**Caregiver (CG) (N=24)**				
	Age (years), mean (SD)		59.58 (11.99)	
	Female sex, n (%)		17 (71)	
	Married or living with a partner, n (%)		21 (88)	
	Years of living in the United States, mean (SD)		23.4 (10.5)	
	Limited English proficiency, n (%)		13 (54)	
	Speaks Chinese or Mandarin at home, n (%)		22 (92)	
	Has difficulty paying for basics, n (%)		11 (46)	
**Care partner (CP) (N=24)**				
	Age (years), mean (SD)		81.38 (8.65)	
	Female sex, n (%)		13 (54)	
	CP and CG live together, n (%)		16 (67)	
	**Relationship to CG**			
		Spouse, n (%)	7 (29)	
		Child, n (%)	16 (67)	
		Other relative or friend, n (%)	1 (4)	
	ADL^a^ score, mean (SD)		11.54 (9.47)	
	IADL^b^ score, mean (SD)		20.08 (5.64)	

^a^ADL: activity of daily living.

^b^IADL: instrumental activities of daily living.

### Feasibility

Three indicators were used to assess feasibility: (1) follow-up rate, (2) curriculum completion rate, and (3) user activities. Out of 24 participants who were enrolled at baseline, a total of 23 completed the intervention and follow-up survey, with a retention rate of 96%. The backend database revealed that out of the 23 participants in the follow-up, the curriculum completion rate ranged from 8% to 100% with a mean value of 67%. Participants’ total read counts of program components ranged from 5 to 154 with a mean of 54. Participants’ total reading minutes ranged from 1 to 7196 minutes with a mean of 465 minutes (see Multimedia Appendix 3 for a table on each participant’s read count, reading minutes, and completion rates). Out of 23 participants, 6 (27%) completed less than one-third of the WECARE program, 4 (17%) completed one-third to two-thirds of the program, and 13 (56%) completed more than two-third of the program, suggesting most participants had a high level of user engagement (see Multimedia Appendix 3).

### Acceptability

Acceptability was assessed with four indicators, including (1) user satisfaction and (2) perceived usefulness of overall WECARE program, and (3) perceived usefulness and (4) user activity on the weekly program. Table 3 depicts user satisfaction of the WECARE program, and the mean total score was 32.52 out of the possible range of 5 to 35. Table 4 illustrates the perceived usefulness of the WECARE program, and the mean total score was 23.17 out of the possible range of 5 to 25. Table 5 details users’ perceived usefulness of each week’s program, and the score ranged from 4.35 to 4.65 out of a range of 1 to 5. Table 5 also lists the mean read counts and reading minutes by weekly program. The read counts for weekly program ranged from 6.5 to 10.6 times; the average total reading minutes of 23 participants for weekly program ranged from 40 to 132 minutes, with a big variation between weeks, see Multimedia Appendix 3 for mean weekly read counts and mean weekly reading minutes.

**Table 3:** User satisfaction (N=23).

Item	Mean (SD)
1. It was easy to use	4.87 (0.34)
2. It was useful for me	4.70 (0.47)
3. The time needed for the program was appropriate	4.65 (0.49)
4. It was boring to use (reversed score)	4.48 (0.95)
5. It was fun to use	4.48 (0.67)
6. I would recommend it to others	4.70 (0.47)
7. Overall, I’m satisfied with the program	4.65 (0.49)
Total score	32.52 (2.54)

**Table 4:** Perceived usefulness (N=23).

Item	Mean (SD)
1. WECARE^a^ has helped me understand Alzheimer disease better	4.74 (0.45)
2. WECARE has motivated me to become a better caregiver	4.78 (0.42)
3. WECARE has helped me become a better caregiver	4.65 (0.49)
4. WECARE has helped me better manage stress and improve my psychosocial well-being	4.48 (0.67)
5. WECARE has helped me to better prepare the upcoming journey of caregiving	4.52 (0.51)
Total score	23.17 (1.99)

^a^WECARE: Wellness Enhancement for Caregivers.

**Table 5:** Perceived usefulness and engagement by weekly program (N=23).

	Usefulness (range 1-5), mean (SD)	Read counts by week, mean (SD)	Reading minutes by week, mean (SD)
1. Week 1: Dementia facts and knowledge	4.52 (0.59)	10.6 (8.03)	132.1 (465.15)
2. Week 2: Caring for patients with dementia	4.65 (0.49)	8.3 (5.89)	113.5 (400.76)
3. Week 3: Effective communication	4.43 (0.66)	7.3 (6.46)	22.5 (31.38)
4. Week 4: Problem-solving in caregiving	4.35 (0.65)	7.1 (5.53)	40.4 (118.16)
5. Week 5: Stress reduction and depression prevention	4.35 (0.65)	8.7 (7.15)	49.3 (135.38)
6. Week 6: Becoming a healthy caregiver	4.39 (0.72)	6.5 (5.88)	40.3 (126.20)
7. Week 7: Course summary and local resources	4.57 (0.59)	8.2 (5.48)	66.5 (210.90)
8. Three group meetings online	4.39 (0.78)	—^a^	—

^a^Not available.

### Preliminary Efficacy

The intervention efficacy was assessed with pre-post differences of 4 psychosocial outcomes: depressive symptoms, caregivers’ burden, life satisfaction, and social support. Table 6 lists the results of the pre-post differences and effect sizes of these measures. Despite a small sample size, 3 out of 4 outcomes had statistically significant differences. Specifically, depressive symptoms decreased from 5.74 at baseline to 3.35 at follow-up; the effect size was −0.89. Caregiving burden decreased from 25.78 to 21.91, and the effect size was −0.48. Life satisfaction increased from 11.35 to 14.83, and the effect size was 0.55. However, there was no significant change in social support.

**Table 6:** Caregivers’ psychosocial well-being pre- and postintervention comparison (N=23).

Outcome	Baseline, mean (SD)	Follow-up, mean (SD)	Change, mean (95% CI)	Effect size	*P* value
Depressive symptoms (range 0-10)	5.74 (2.56)	3.35 (2.72)	−2.39 (−3.56 to −1.23)	−0.89 (−1.37 to −0.40)	<.001^a^
Caregiving burden (range 0-48)	25.78 (7.19)	21.91 (6.69)	−3.87 (−7.38 to −0.36)	−0.48 (−0.90 to −0.04)	.03^b^
Life satisfaction (range 0-30)	11.35 (6.66)	14.83 (7.11)	3.48 (0.73 to 6.23)	0.55 (0.10 to 0.98)	.02^c^
Social support (range 0-30)	14.78 (5.15)	13.96 (6.39)	−0.83 (−3.39 to 1.74)	−0.14 (−0.55 to 0.27)	.51

^a^*P*<.005.

^b^*P*<.05.

^c^*P*<.01.

## Discussion

### Principal Findings

Our data strongly suggest that WECARE was a feasible and acceptable intervention in Chinese American dementia caregivers; it also demonstrated preliminary efficacy in improving participants’ psychosocial well-being. First, the intervention was feasible. A total of 24 participants enrolled in the study and 23 completed the intervention with a retention rate of 96%. The backend database that tracked user activities showed that the mean curriculum completion rate was 67%; in other words, on average participants had completed 67% of all 40 multimedia papers in the 7-week program. The mean read counts was 57, and the mean total reading minutes was 465 minutes, suggesting a good level of user engagement. Second, the intervention had good acceptability. The follow-up survey indicated that participants reported high levels of user satisfaction (32 out of 35), high levels of perceived usefulness of the intervention (23 out of 25), and high levels of perceived usefulness of weekly programs (4.3-4.5 out of 5). Third, the intervention demonstrated preliminary efficacy. The pre-post analysis of psychosocial outcomes revealed that, despite a small sample size, 3 out of 4 health outcomes, that is, depressive symptoms, caregivers’ burden, and life satisfaction, had statistically significant changes after the intervention, and the effect sizes ranged from 0.55 to 0.89. However, perceived social support for caregiving remained unchanged.

### Data Interpretation

To the best of our knowledge, the WECARE represents the first mHealth intervention for Chinese American dementia caregivers and one of the first for immigrant and racial minority dementia caregivers. The results from the trial were comparable to earlier in-person interventions for Chinese American dementia caregivers [28,29] and other in-person interventions for other racial or ethnic minority dementia caregivers [54,55]. It is worth noting that the demographic characteristics of the participants in this study were comparable to earlier community-based studies on Chinese American dementia caregivers [21,29]. As underserved Chinese American dementia caregivers have high rates of psychosocial distress due to high intensity, duration, and complexity of caregiving, the success of the WECARE suggests a promising solution to deliver effective mHealth interventions to address the needs of this vulnerable group.

The feasibility, acceptability, and initial efficacy demonstrated in this study could be attributed to the following strengths of the WECARE. First, the curriculum of the WECARE was developed based on evidence-based interventions [50,56]. Second, the culturally tailored program components were developed using community-engaged user-centered design principles [57]. We worked closely with our community partners through an iterative process of design, test, and revise. The resulting WECARE program consists of 40 interactive multimedia articles that reflect Chinese American family values and social norms of caregiving; it also includes relevant information and resources urgently needed by these linguistically isolated caregivers. Third, the WECARE was delivered via WeChat, a popular social media app used frequently by participants; it was easy to adopt for continuous use [37].

We noted that despite significant improvement in 3 out of the 4 health outcomes (depressive symptoms, caregiving burden, and life satisfaction), participants still reported high levels of depressive symptoms (mean 3.35) and caregiving burden (mean 21.91) at the follow-up, suggesting elevated psychosocial distress despite the intervention. More resources and continuous support are needed to meet the needs of this vulnerable population.

One of the 2 secondary outcomes for efficacy evaluation, perceived social support for caregiving, did not change significantly after the intervention. There were 2 possible explanations. One, it might be that the sample size was too small to detect the change. Two, the WECARE had minimal effect on improving perceived social support in caregiving, even though 3 moderated online meetings were organized and participants could use the built-in functions in WeChat like group chat and private chat to initiate additional contacts. Social support requires long-term trust building and tangible support to address daily needs. A 7-week online program with limited interactions might not be the most effective approach to improve social support.

An important feature of the WECARE was its backend database that automatically sent program components and tracked user activities, including whether and when an article was opened, how many times it was read, and for how long. User activity data such as curriculum completion rate, total read counts, and total reading minutes could be used as objective measure of user engagement. These data also complemented the self-report survey data on user satisfaction and perceived usefulness to provide a more comprehensive understanding of the program’s feasibility and acceptability and inform further revision of the intervention. For example, high levels of user engagement tracked by the backend database and high levels of perceived usefulness reported in the follow-up survey for a particular week’s program might indicate its good acceptability; otherwise, it might suggest the need for further revision.

### Limitations

This pilot study has several limitations. First, there was no control group, so we could not affirm whether the changes in health outcomes observed in this pre-post trial were a result of the intervention only instead of testing effects or other factors. The main purpose of the pilot study was to assess the feasibility, acceptability, and preliminary efficacy of the WECARE; thus, future research would need to test its efficacy through a rigorous randomized controlled trial. Second, all acceptability measures and health outcome measures were based on self-report, there were potential self-report biases. Third, the follow-up survey was administered 2-3 weeks after the intervention, so we were not able to observe the long-term effect of WECARE. A future study with long-term follow-ups is needed. Fourth, though the intervention retention rate was 96% with only 1 participant dropped out, the curriculum completion rate was suboptimal with 67%, suggesting some participants stayed in the intervention but did not complete the entire curriculum. However, these numbers were higher or comparable to other mHealth interventions for caregivers or other WeChat-based interventions [35,36]. Fifth, our sample size was rather small, so we were not able to perform any stratified analysis or examine the independent relationships between the intervention effects and key covariates such as demographics and user engagement, for example, if the intervention was more effective in some demographic groups. A future study with a larger sample size would be able to address this limitation.

### Conclusions

The WECARE pilot study demonstrated that this WeChat-based intervention demonstrated a high level of feasibility and acceptability; it also showed promising efficacy in improving psychosocial well-being in Chinese American family caregivers of persons with dementia. It reduced participants’ depressive symptoms, decreased caregiving burden, and increased life satisfaction but had no effect on perceived social support for caregiving. Our next step is to conduct a randomized controlled trial with a larger sample and long-term follow-up to further test WECARE’s efficacy and effectiveness. Based on the promising results from this study, we call for more research on culturally tailored and digitally delivered interventions for immigrant and racial or ethnic minority family caregivers of persons with dementia.

## Appendix

Multimedia Appendix 1 WECARE project baseline and follow-up survey.

Multimedia Appendix 2 WECARE intervention frontend and backend screenshots.

Multimedia Appendix 3 User activities tracked by the backend database.

## Abbreviations

ADL: activity of daily living
CES-D: Center for Epidemiologic Depression Scale
IADL: instrumental activities of daily living
mHealth: mobile health
SSC: Social Support Scale
SWLS: Satisfaction With Life Scale
WECARE: Wellness Enhancement for Caregivers
ZBI: Zarit Burden Interview

## Data Availability

This pilot study has a sample size of 24 only, and all participants were underserved Chinese American dementia caregivers. Even deidentified day poses potential risks of tracing back to some participants. Per our institutional review board requirement, we do not submit the data to a repository. Any fellow researcher interested in this data set may contact the corresponding author directly.
