# Supplementary material for: A WeChat-based Intervention, Wellness Enhancement for Caregivers (WECARE), for Chinese American Dementia Caregivers: Pilot Assessment of Feasibility, Acceptability, and Preliminary Efficacy
Source: JMIR Aging. 2023 Apr 5;6:e42972. doi: 10.2196/42972 (PMC10131589; doi:10.2196/42972)
Supplement: Multimedia Appendix 1 [file aging_v6i1e42972_app1.pdf]

Appendix: WECARE Baseline and Follow-up survey

A. Demographics of caregiver (Baseline only)

1. What's your age? _________Years
2. What's your gender?
   - o Male
   - o Female
3. What the highest level of your education?
   - o Elementary school or middle school
   - o High school
   - o College or above
4. Have you been educated in the U.S.?
   - o Yes
   - o No

   4.a (If yes), how many years? ____________years
5. What's your marital status?
   - o Married or living with a partner
   - o Single, divorced, separated, or widowed
6. How many children do you have?
   - o 0
   - o 1
   - o 2
   - o 3
   - o 4+
7. What's your current living arrangement?
   - o Live with family
   - o Live with friends or roommates
   - o Live alone

   7a. (If live alone) How many people live in your house? ____________
8. What's your employment status?
   - o Employed fulltime
   - o Employed part-time
   - o Retired
   - o Not employed
9. How many years have you lived in the United States?
10. Do you have health insurance?
    - o Medicare
    - o Medicaid
    - o Obamacare
    - o Private insurance
    - o Others
    - o I don't have health insurance
11. Do you have a primary care doctor or a doctor you see regularly?

- Yes
- No

12. How well do you speak English?
    - Not all
    - Not well
    - A little
    - Somehow fluent
    - Very fluent
13. What's the primary language spoken at your house?
    - English
    - Simplified Chinese
    - Traditional Chinese
    - other_____
14. Do you have difficulty paying for basics (e.g., rent/mortgage, utility bills, groceries)?
    - Not difficult at all
    - Somewhat difficult
    - Very difficult
15. How would you rate your overall health?
    - Excellent
    - Good
    - Fair
    - Poor
16. How often do you use WeChat? (Multiple times a day, once a day, several days a week, once a week or less)
    - Multiple times a day
    - Once a day
    - Several days a week
    - Once a week or less
17. Do you have the following mobile devices (select all that apply):
    - Desktop computer
    - Laptop, tablet (i.e., iPad)
    - Smartphone
    - Wearable device (i.e., smartwatch, Fitbit)

B. Demographics of care-partner (Baseline only)

1. How old is the care partner? ___________years
2. What's the gender your care partner?
    - Male
    - Female
3. What's the diagnosis of your care partner?
    - Alzheimer's disease
    - Other dementias: ________
    - Unclear diagnosis

    3a. When did your care partner get diagnosed? (Year ___, month____)

4. What’s your relationship with the care partner?
   - Child
   - Grandchild
   - Nephew, niece or other relative or friend_____
5. Does the care partner have insurance?
   - Yes
   - No
6. Do you live with the care partner?
   - Yes
   - No

   6.1 (If no) how far are you from your place to the care-partner? _____hours travel time.

   6.2 Does care-partner live in a nursing home or senior health center? (yes/no)

   6.2.(a) (if yes) how long has the care partner live in the nursing home or senior health center? _______

   6.2.(b) (if no) Does care-partner go to an adult care? (yes/no)

   6.2b-1 (if yes), how often for a week? (1,2,3,4, >=5).

   6.2b-2 (if no) Do you use in-home care services (yes/no)

   6.2b-2a (if yes), how often for a week? (1,2,3,4, >=5).
7. How long have you taken care of the care-partner (___years__months)
8. On average, how many hours a week do you spend with care-partner? ___hours
9. Other than you, are there are family members who also take care of the care-partner? (Yes/no)

   9a. (If yes), how many_____
10. How often do you receive help for caring for care-partner?
   - Always
   - Often
   - Sometimes
   - Occasionally
   - Never

C1. Care-partner’s Activities of Daily Living (ADL) (Baseline only)

	Independent	Needs help	Dependent	Cannot do
**Bathing**				
**Dressing**				
**Grooming**				
**Mouth care**				
**Toileting**				
**Transferring bed/chair**				
**Walking**				
**Climbing stairs**				
**Eating**				

C2. Instrumental Activities of Daily Living (IADL) (Baseline only)

	Independent	Needs help	Dependent	Cannot do
**Shopping**				
**Cooking**				
**Managing medications**				
**Using the phone and looking up numbers**				
**Doing housework**				
**Doing laundry**				
**Driving or using public transportation**				
**Managing finances (e.g, pay bills)**				

D. Caregiver's depressive symptoms (CESD, 10 items) (Baseline and Follow-up)

Please tell us how often you have felt this way during the **past 7 days**.

A. I felt depressed	Yes	No
B. I felt that everything I did was an effort	Yes	No
C. My sleep was restless	Yes	No
D. I was happy (R)	Yes	No
E. I felt lonely	Yes	No
F. People were unfriendly	Yes	No
G. I enjoyed life (R)	Yes	No
H. I felt sad	Yes	No
I. I felt that people disliked me	Yes	No
J. I could not get going	Yes	No

E. Life satisfaction (Baseline & Follow-up)

	Strongly agree	Agree	Slightly agree	Neither agree nor disagree	Slightly disagree	Disagree	Strongly disagree
In most ways, my life is close to my ideal							

The conditions of my life are excellent							
I'm satisfied with my life							
So far I have gotten the important things I want in life							
If I could live my life over, I would change almost nothing							

F. Caregiving Burden (ZBI 12 items) (Baseline & Follow-up)

Do you feel….?	Never	Occasionally	Some-times	Often	Nearly almost
That because of the time you spend with your relative that you don't have enough time for yourself?					
Stressed between caring for your relative and trying to meet other responsibilities (work/family)?					
Angry when you are around your relative?					
That your relative currently affects your relationship with family members or friends in a negative way?					
Strained when you are around your relative?					
That your health has suffered because of your involvement with your relative?					
That you don't have as much privacy as you would like because of your relative?					
That your social life has suffered because you are caring for your relative?					
That you have lost control of your life since your relative's illness?					
Uncertain about what to do about your relative?					
You should be doing more for your relative?					
You could do a better job in caring for your relative?					

G. Self-Care: REACH Composite Measure (Baseline only)

These questions will be about **your own** health.

1. In the **past month**, have you lost or gained weight without meaning to? (Baseline & Follow-up)

- No
- Yes

2. In the **past 6 months**, have you missed any scheduled doctor's appointments?
    - No
    - Yes
3. In the **past 6 months**, have you found that you had the time to see your doctor when you thought you should?
    - No
    - Yes
4. In the **past 6 months**, have you found that you were able to slow down and get enough rest when you were sick?
    - No
    - Yes
5. In the **past year**, have you seen your doctor for a routine check-up?
    - No
    - Yes
6. In the past year, have you had your eyesight checked?
    - No
    - Yes
7. In the **past year**, have you had your hearing checked?
    - No
    - Yes
8. In the **past year**, have your teeth/dentures been examined by a dentist?
    - No
    - Yes
9. In the **past year**, have you had a flu shot?
    - No
    - Yes
10. In the **past year**, have you had your blood pressure checked?
    - No
    - Yes

11a. **In the past year: MALE ONLY**
Have you had your prostate examination?
- No
- Yes

11b. **In the past 2 years: FEMALE ONLY**
Have you had a mammogram or Pap smear?
- No
- Yes

H. Social Support: REACH Composite Score (Baseline and follow-up)
Next, I will ask you questions about support you receive from those around you.

1. Do you have someone to comfort you, listen to your feelings, etc.?
    - Never
    - Once a While
    - Fairly Often
    - Very Often
2. Do you feel isolated from your family and friends?
    - Never
    - Once a While
    - Fairly Often
    - Very Often
3. Overall, how satisfied have you been in the past month with the help you've received from family members, friends, or neighbors?
    - Not at all
    - A little
    - Moderately
    - Very
4. How many relatives, friends, neighbors, other than (Care Partner), do you see or hear from at least once a month?
    - None
    - 1-2
    - 3-4
    - >= 5
5. How many relatives, friends, neighbors, other that (Care Partner), do you feel you can call on for help with chores, transportation, etc.?
    - None
    - 1-2
    - 3-4
    - >= 5
6. When other people you know have an important decision to make, do they talk to you about it?
    - Never
    - Once a While
    - Fairly Often
    - Very Often
7. In the past month, how often has someone, such as a family member, friend, or neighbor, other than (Care Partner), provided transportation or pitched in to help you do something that needed to get done, like household chores or yard work and/or helped you with shopping?
    - Never
    - Once a While
    - Fairly Often

- Very Often

8. In the past month, overall, how satisfied have you been with the help you have received with transportation, housework, yard work and shopping?
    - Not at all
    - A little
    - Moderately
    - Very

9. In the past month, how often has someone been there with you (physically) in a stressful situation, provided comfort to you, or expressed concern about your well-being?
    - Never
    - Once a While
    - Fairly Often
    - Very Often

10. In the past month, how satisfied have you been with the support, comfort, interest and concern you have received from others?
    - Not at all
    - A little
    - Moderately
    - Very

I. Problem Behaviors: Questions assessing domains of the Revised Memory Behavior Problem Checklist (Baseline only)
The final set of questions refers to your care partner's health.

1. In the past six months, have you seen any cognitive improvement in care partner overall?
    - No
    - Yes
    - Unknown
2. In the past six months, have you seen any improvement in care partner mood overall?
    - No
    - Yes
    - Unknown
3. In the past six months, have you seen any behavioral improvement overall?
    - No
    - Yes
    - Unknown

I. Problem Behaviors: Questions assessing domains of the Revised Memory Behavior Problem Checklist (Follow-up only)
The final set of questions refers to your loved one's health.

1. In the past 8 weeks, have you seen any cognitive improvement in care partner overall?
    - No

- Yes
- Unknown

2. In the past 8 weeks, have you seen any improvement in (Care Partner's) mood overall?
   - No
   - Yes
   - Unknown
3. In the 8 weeks, have you seen any behavioral improvement overall?
   - No
   - Yes
   - Unknown

K. **The following questions about your experience on the WECARE program** (Follow-up only)

K.1. User satisfaction

	Strongly disagree (1)	Disagree (2)	Neutral (3)	Agree (4)	Strongly agree (5)
1) It was easy to use					
2) It was useful for me					
3) The time needed for the program was appropriate					
4) It was boring to use					
5) It was fun to use					
6) I would recommend it to others					
7) Overall, I'm satisfied with the program					

K.2. Perceived usefulness

	Strongly disagree (1)	Disagree (2)	Neutral (3)	Agree (4)	Strongly agree (5)
1) WECARE has helped me understand Alzheimer's disease better					
2) WECARE has motivates me to become a better caregiver					
3) WECARE has helped me become a better caregiver					
4) WECARE has helped me better manage stress and improve my psychosocial wellbeing					
5) WECARE has helped me to better prepare the upcoming journey of caregiving					

L. **The following questions about your feedback on each week of the curriculum (Follow-up only)**

	Easy to use			Useful for me		
	No	Neutral	Yes	No	Neutral	Yes
1) Week 1: Program overview and introduction						
2) Week 2: Caring for ADRD patients						
3) Week 3: Effective communication						
4) Week 4: Problem solving in caregiving						
5) Week 5: Stress reduction and depression prevention						
6) Week 6: Becoming a healthy caregiver						
7) Week 7: Course summary						
8) 3 group meetings online						
