# Supplementary material for: A WeChat-based Intervention, Wellness Enhancement for Caregivers (WECARE), for Chinese American Dementia Caregivers: Pilot Assessment of Feasibility, Acceptability, and Preliminary Efficacy
Source: JMIR Aging. 2023 Apr 5;6:e42972. doi: 10.2196/42972 (PMC10131589; doi:10.2196/42972)

Appendix: Selected WECARE program screenshots

Figure 1: Sample screenshots of user interface

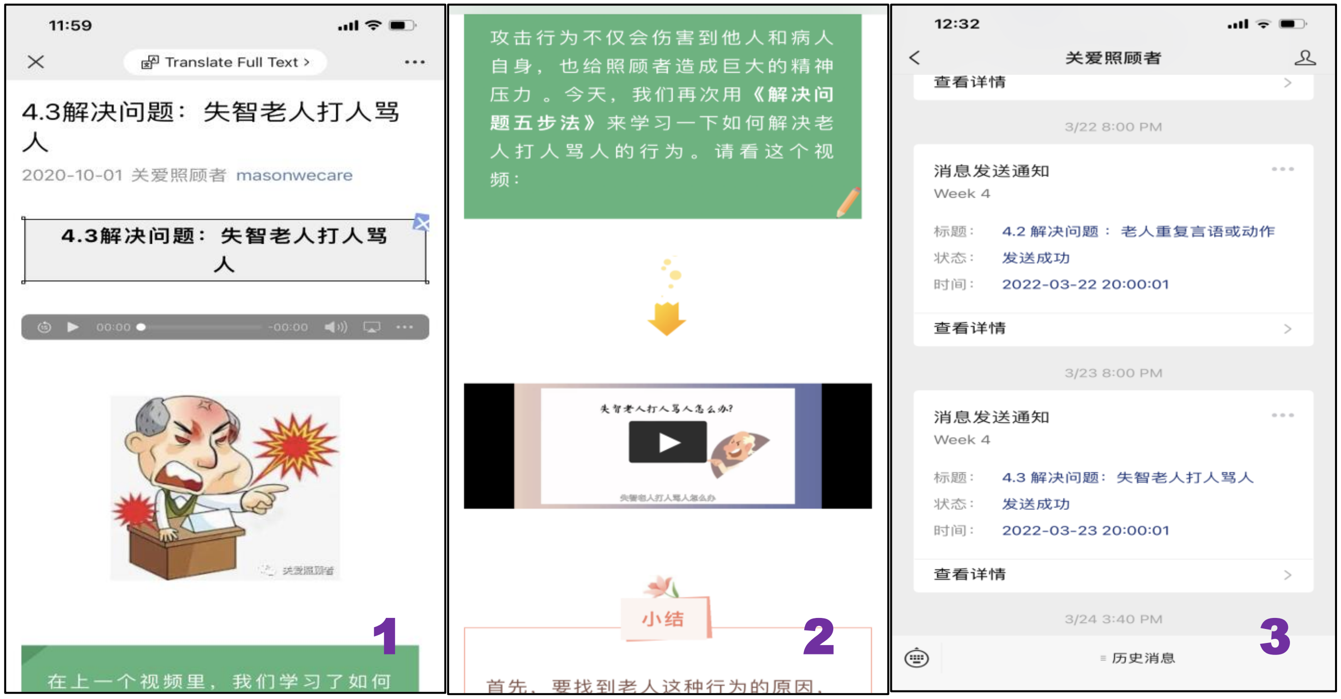

1. Multimedia article with audio recording on how to deal with angry behaviors of dementia patients
2. Short video clip as a case study to explain the angry behaviors of dementia patients
3. Push notification & progress summary

Figure 2: WECARE backend database: Scheduled delivery system

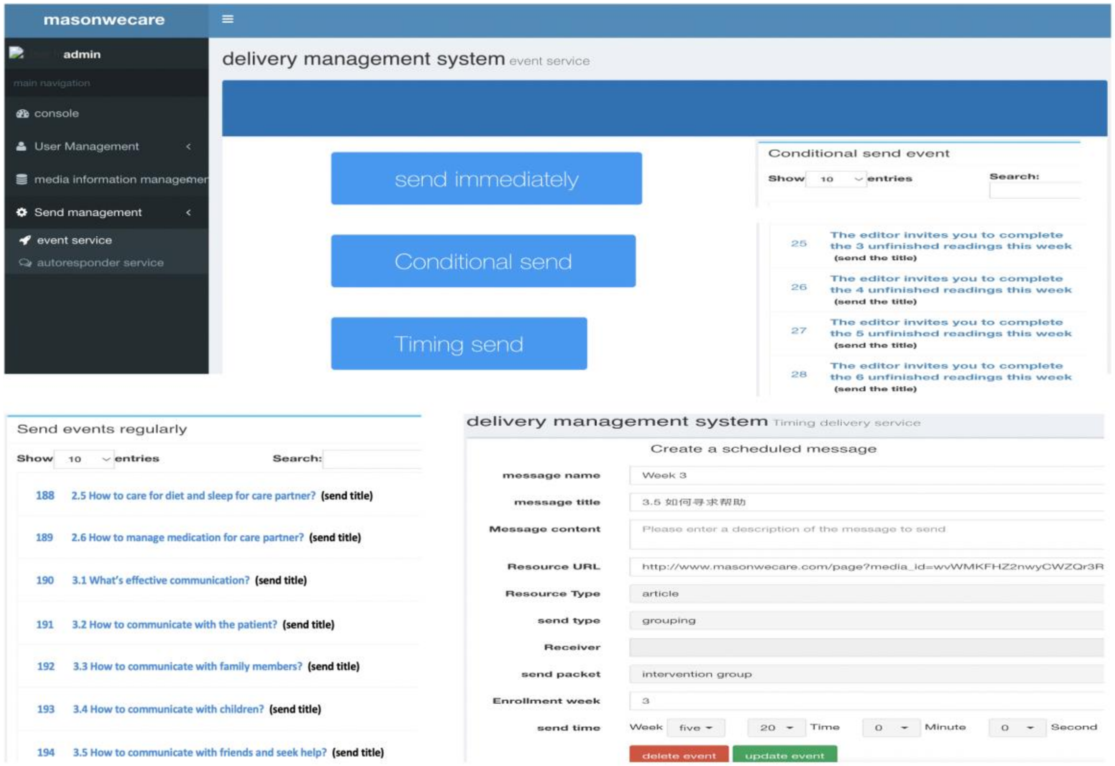

Figure 3: WECARE backend database: User management system

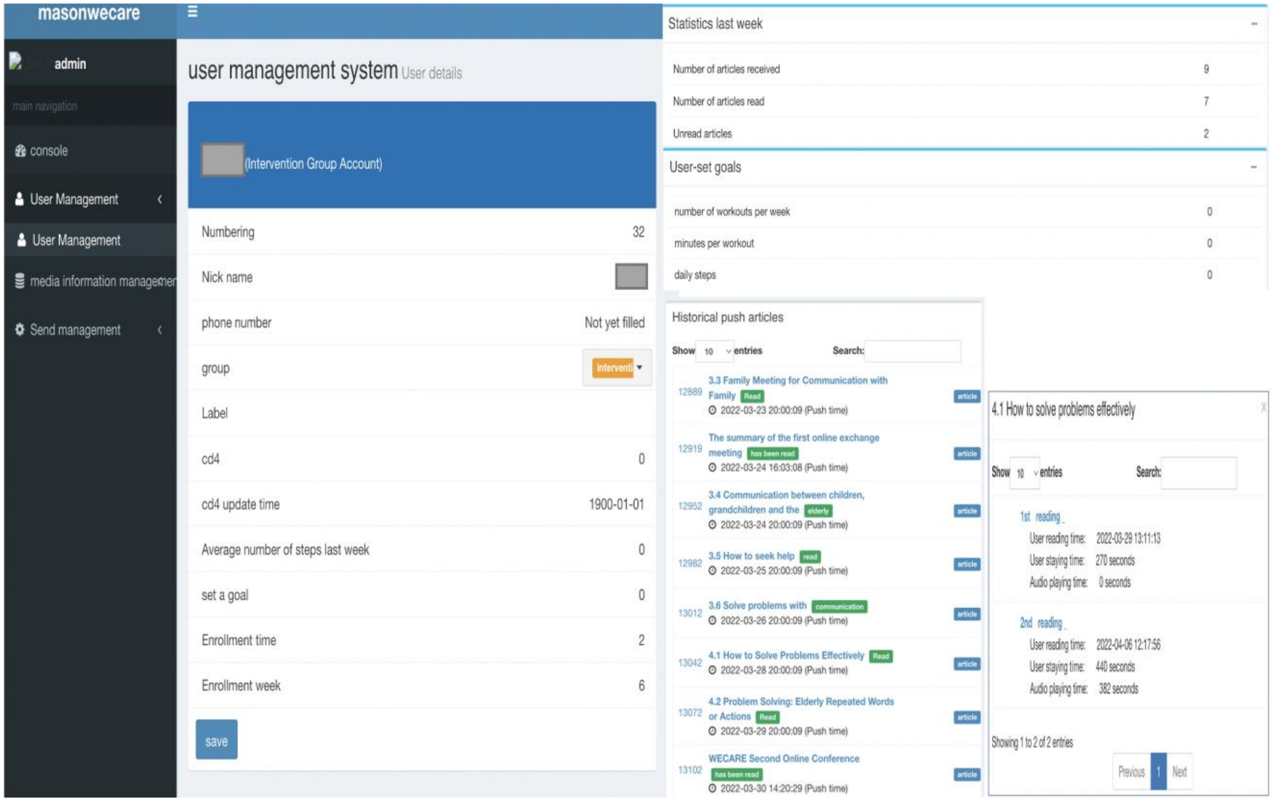

Supplement: Multimedia Appendix 2 [file aging_v6i1e42972_app2.pdf]
