# Supplementary material for: A WeChat-based Intervention, Wellness Enhancement for Caregivers (WECARE), for Chinese American Dementia Caregivers: Pilot Assessment of Feasibility, Acceptability, and Preliminary Efficacy
Source: JMIR Aging. 2023 Apr 5;6:e42972. doi: 10.2196/42972 (PMC10131589; doi:10.2196/42972)
Supplement: Multimedia Appendix 3 [file aging_v6i1e42972_app3.docx]

**Appendix 3: User activities tracked by the backend database**

Table 1. Participant’s read counts, reading minutes, and course completion rates

|  | **Total read count^1^** | **Total reading minutes^2^** | **Course completion^3^** |
| --- | --- | --- | --- |
| **1** | 6 | 38 | 0.08 |
| **2** | 5 | 1 | 0.13 |
| **3** | 11 | 29 | 0.13 |
| **4** | 9 | 11 | 0.18 |
| **5** | 25 | 28 | 0.26 |
| **6** | 29 | 86 | 0.32 |
| **7** | 26 | 31 | 0.34 |
| **8** | 53 | 250 | 0.57 |
| **9** | 37 | 17 | 0.66 |
| **10** | 35 | 48 | 0.68 |
| **11** | 58 | 81 | 0.76 |
| **12** | 88 | 184 | 0.84 |
| **13** | 78 | 194 | 0.84 |
| **14** | 64 | 438 | 0.87 |
| **15** | 138 | 520 | 0.89 |
| **16** | 51 | 48 | 0.92 |
| **17** | 72 | 307 | 0.95 |
| **18** | 154 | 7196 | 0.95 |
| **19** | 62 | 52 | 1.00 |
| **20** | 95 | 487 | 1.00 |
| **21** | 58 | 86 | 1.00 |
| **22** | 81 | 390 | 1.00 |
| **23** | 68 | 164 | 1.00 |

1) Read count: Number of times WECARE was opened

2) Reading minutes: Time (in minutes) spent on WECARE

3) Course completion: Percentage of program components were read.

Low engagement (completed less than 33%): n=6 (26.09%)

Medium engagement (completed 33%~68% of program): n=4 (17.39%)

High engagement (completed more than 68% of the program): n=13 (56.52%)

Figure 1: Read counts and reading minutes by week (n=23)

|  | Weekly read counts and reading minutes in average |  |
| --- | --- | --- |
|  |  |  |
| Week | Reading count | Reading minutes |
| W1 | 10.6 | 132.1 |
| W2 | 8.3 | 113.5 |
| W3 | 7.3 | 22.5 |
| W4 | 7.1 | 40.4 |
| W5 | 8.7 | 49.3 |
| W6 | 6.5 | 40.3 |
| W7 | 8.2 | 66.5 |
